# Predicting seizure outcome of vagus nerve stimulation using MEG-based network topology

**DOI:** 10.1016/j.nicl.2018.06.017

**Published:** 2018-06-18

**Authors:** Abbas Babajani-Feremi, Negar Noorizadeh, Basanagoud Mudigoudar, James W. Wheless

**Affiliations:** a Department of Pediatrics, University of Tennessee Health Science Center, Memphis, TN, USA; b Le Bonheur Children's Hospital, Neuroscience Institute, Memphis, TN, USA; c Department of Anatomy and Neurobiology, University of Tennessee Health Science Center, Memphis, TN, USA

**Keywords:** Vagus nerve stimulation (VNS), Seizure outcome, VNS efficacy, Magnetoencephalography (MEG), Functional connectivity, Phase-locking value (PLV), Graph measures, Human connectome project (HCP)

## Abstract

Vagus nerve stimulation (VNS) is a low-risk surgical option for patients with drug resistant epilepsy, although it is impossible to predict which patients may respond to VNS treatment. Resting-state magnetoencephalography (rs-MEG) connectivity analysis has been increasingly utilized to investigate the impact of epilepsy on brain networks and identify alteration of these networks after different treatments; however, there is no study to date utilizing this modality to predict the efficacy of VNS treatment. We investigated whether the rs-MEG network topology before VNS implantation can be used to predict efficacy of VNS treatment. Twenty-three patients with epilepsy who had MEG before VNS implantation were included in this study. We also included 89 healthy control subjects from the Human Connectome Project. Using the phase-locking value in the theta, alpha, and beta frequency bands as a measure of rs-MEG functional connectivity, we calculated three global graph measures: modularity, transitivity, and characteristic path length (CPL). Our results revealed that the rs-MEG graph measures were significantly heritable and had an overall good test-retest reliability, and thus these measures may be used as potential biomarkers of the network topology. We found that the modularity and transitivity in VNS responders were significantly larger and smaller, respectively, than those observed in VNS non-responders. We also observed that the modularity and transitivity in three frequency bands and CPL in delta and beta bands were significantly different in controls than those found in responders or non-responders, although the values of the graph measures in controls were closer to those of responders than non-responders. We used the modularity and transitivity as input features of a naïve Bayes classifier, and achieved an accuracy of 87% in classification of non-responders, responders, and controls. The results of this study revealed that MEG-based graph measures are reliable biomarkers, and that these measures may be used to predict seizure outcome of VNS treatment

## Highlights

• We investigated whether rs-MEG network topology can predict efficacy of VNS treatment
• We calculated graph measures based on PLV as a measure of rs-MEG functional connectivity
• The graph measures were significantly heritable and had an overall good test-retest reliability
• The modularity and transitivity were significantly different in responders versus non-responders
• We achieved an accuracy of 87% in classification of non-responders, responders, and controls
• Modularity and transitivity of rs-MEG may be used as clinical biomarkers for prediction of VNS outcome

## 1 Introduction

Vagus nerve stimulation (VNS) is a low-risk surgical option for approximately one-third of patients with epilepsy who continue to seize despite tailored medical therapy (Mohanraj and Brodie, 2006). It has been demonstrated that VNS is effective in reducing seizure frequency by >50% in approximately 50% of patients (responders), though reduction in seizure frequency is <50% in about one half of patients (non-responders) and approximately 25% of patients experienced no measurable benefit from VNS treatment (Englot et al., 2011). Despite the growing application of VNS, it is still not possible to predict which patients will respond to VNS treatment, and the degree of their response. In recent years, resting-state magnetoencephalography (rs-MEG) connectivity analysis has been used in several epilepsy studies to lateralize and localize the seizure onset zone (Nissen et al., 2017), investigate alteration of the brain connectivity patterns from interictal to preictal (Hamandi et al., 2016), and relate alterations of the brain connectivity to duration and severity of epilepsy (Englot et al., 2015). Although MEG has been increasingly utilized in investigation of the brain networks in patients with epilepsy, there is no study to date that utilizes this modality to predict the efficacy of VNS treatment. The aim of this study was to predict VNS seizure outcome using rs-MEG data collected before implantation of VNS.

Graph measures can extract useful features from the brain network (Bullmore and Sporns, 2009). Network topology has been used to characterize structural and functional properties of the brain networks in healthy subjects and patients (Ridley et al., 2015; Garcia-Ramos et al., 2016; Rubinov and Sporns, 2010). The brain of a healthy subject is a complex network and has properties such as balanced integration and segregation; however, the brain of patients with epilepsy is deviated toward regular or random networks, with alterations in integration and segregation characteristics. Previous studies have demonstrated alteration of graph measures in patients with epilepsy compared to healthy control subjects using electroencephalography (EEG) (van Diessen et al., 2016), MEG (Niso et al., 2015), electrocorticography (ECoG) (Vega-Zelaya et al., 2014), and functional MRI (fMRI) (Ridley et al., 2015; Garcia-Ramos et al., 2016; Doucet et al., 2015). Despite the growing applications of network topology in patients with epilepsy, there is only one study that used EEG-based graph measures and investigated the difference in these measures in responders and non-responders to VNS treatment (Fraschini et al., 2014). Moreover, this study did not investigate the network topology of the patients before implantation of the VNS, in the interests of finding a biomarker that could predict which patients will respond to VNS.

We investigated whether individual differences in the network topology (i.e. CPL, transitivity, and modularity), derived from rs-MEG functional connectivity, have a biological basis by establishing them as heritable traits. To establish clinical applicability of the network topology as biomarkers, these biomarkers have to relate to naturally occurring variation, such as those caused by genetic factors. Therefore, demonstrating sensitivity of a network topology biomarker to genetic influences provides some external, genetic validity to the biomarker. Furthermore, if it is demonstrated that a network topology biomarker is heritable, and the value of this biomarker is significantly different between VNS-responders and non-responders, this motivates further research into specific candidate genes, or sets of genes, that may affect the response to VNS treatment. Heritability of rs-MEG functional connectivity has been observed in a few studies (Demuru et al., 2017; Colclough et al., 2017); however, these studies did not investigate heritability of the network topology. We address this deficiency in the current study.

Previous studies reported different characteristics of EEG in responders and non-responders to VNS treatment (Fraschini et al., 2014; Fraschini et al., 2013; Bodin et al., 2015). Using EEG data acquired after implantation of VNS, Fraschini et al. demonstrated that patients responding to VNS represent a brain network reorganization toward a more integrated architecture (Fraschini et al., 2014; Fraschini et al., 2013). A recent study utilized resting-state functional MRI (rs-fMRI) data pre-VNS implantation and found an association of greater VNS efficacy with larger connectivity between the thalami to the anterior cingulate cortex (ACC) and left insula (Ibrahim et al., 2017). These studies provided some evidence that fMRI and EEG can be utilized to detect the differences in the brain networks between VNS responders and non-responders, and ultimately to predict which patient may respond to VNS treatment. Considering this evidence, and the fact that MEG has a superior temporal resolution compared to fMRI and is less affected by the volume-conductor effect compared to EEG, it is expected that the rs-MEG connectivity analysis may provide a reliable prediction of VNS seizure outcome.

In this study, we developed and validated a machine learning approach to predict the VNS seizure outcome using the network topology derived from rs-MEG data, acquired before implantation of VNS. Our hypothesis was that responders and non-responders would have a different network topology, and that this difference could be demonstrated using rs-MEG data acquired before implantation of VNS. In addition to VNS responders and non-responders, we included healthy subjects (controls) who underwent three sessions of rs-MEG data collection from the Human Connectome Project (HCP) (Larson-Prior et al., 2013). The rs-MEG datasets of controls were used to: 1) demonstrate reliability and heritability of the rs-MEG graph measures as potential clinical biomarkers; and 2) compare the network topology of controls with that of VNS responders and non-responders.

## 2 Methods

### 2.1 Participants

#### 2.1.1 Patients

A consecutive series of 121 patients with drug resistant epilepsy who underwent VNS implantation at the Le Bonheur Children's Hospital (LCH) from 2011 to 2017 were retrospectively selected for this study (Table 1). The International League Against Epilepsy established the definition of drug resistant epilepsy as “failure of adequate trials of two tolerated and appropriately chosen and used antiepileptic drug (AED) schedules (whether as monotherapies or in combination) to achieve sustained seizure freedom” (Kwan et al., 2010). The inclusion criteria were patients: (i) who underwent three five-minute sessions (runs) of rs-MEG data collection within one month before VNS implantation; (ii) whose MEG data were not contaminated with artifacts generated by orthodontic devices, ventriculoperitoneal (VP) shunt, and environmental noise; and (iii) who had at least one year follow-up after VNS implantation. Of these 121 patients, 23 patients (11.3 ± 6.9 (mean ± SD) years, 13 female) met inclusion criteria. It is noteworthy that data of 21 patients were contaminated by artifacts.

**Table 1:** Demographic and clinical data for patients.

	Responder	Non-responder	*P*-value
Number of patients (n)	14	9	–
Female (n, %)	7 (50%)	6 (67%)	0.25^b^
Sedated^a^ (n, %)	7 (50%)	5 (56%)	0.32^b^
Age at seizure onset (y, mean ± SD)	4.1 ± 4.6	6.1 ± 7.4	0.44^c^
Duration of epilepsy before receiving VNS (y, mean ± SD)	7.1 ± 5.2	5.4 ± 4.8	0.45^c^
Age at VNS implantation (y, mean ± SD)	11.2 ± 5.4	11.4 ± 9.3	0.93^c^
Follow-up (month, min, max (mean ± SD))	13, 74 (35.0 ± 20.5)	12, 46 (23.4 ± 12.4)	0.16^c^
Pre-VNS seizure frequency (per day, median, IQR)	1.2, 4.9	3.0, 20.7	0.64^d^
Post-VNS seizure frequency (per day, median, IQR)	0.1, 0.4	3.0, 18.3	0.0096^d^
Seizure reduction (%, median, IQR)	85.6%, 35.7%	0.0%, 17.1%	0.000075^d^
Generalized epilepsy (n, %)	4 (29%)	3 (33%)	0.34^b^
Presence of an MRI lesion (n, %)	6 (43%)	3 (33%)	0.31^b^

IQR, interquartile range; SD, standard deviation; y, year.

a 12 patients were sedated for MEG study under general anesthesia.

b *P*-value was calculated using the Fisher exact test.

c P-value was calculated using the *t*-test.

d P-value was calculated using the Mann Whitney test.

All patients were implanted with VNS supplied by LivaNova Inc. (Houston, TX, USA). The VNS stimulation parameters were individually adjusted to achieve optimal efficacy with minimal side effects. Patients were assigned to two groups, VNS responders and non-responders, based on the alteration of the seizure frequency from baseline (i.e. before VNS implantation) to their last follow-up visit. The seizure frequency was determined over one month at both baseline and the last follow-up visit. Reduction in seizure frequency was 50% or more in responders and <50% in non-responders. Twelve patients (approximately 50% of patients in each of the VNS responders and non-responders group) were sedated for MEG study under general anesthesia induced by propofol injection, and maintenance achieved by a propofol infusion rate of 166 μg/kg/min. Eleven patients did not receive sedation during MEG data collection. MEG data from patients were collected as part of a standard of care clinical procedure at the LCH. The decision as to whether or not to collect MEG data under sedation was made clinically. Overall, young and/or developmentally delayed patients who could not follow instructions for MEG data collection were sedated. The study was approved by the Institutional Review Board (IRB) of the University of Tennessee Health Science Center.

#### 2.1.2 Controls

Eighty-nine healthy control subjects (28.6 ± 3.9 (mean ± SD) years, 41 female) from the Human Connectome Project (HCP) who performed three six-minute runs of rs-MEG were included in this study (Larson-Prior et al., 2013). Of these subjects, there were 19 monozygotic (MZ) and 13 dizygotic (DZ) complete twin pairs (Table 2). The HCP, led by Washington University, aimed to characterize human brain function and connectivity in a large number of twins and their siblings (Van Essen et al., 2012). The HCP subjects are young adult healthy individuals who are free from severe neurodevelopmental (e.g. autism), neuropsychiatric (e.g. depression), or neurologic (e.g. epilepsy) disorders.

**Table 2:** Demographic data for healthy control subjects.

Number of subjects	89
Age (year, mean ± SD)	28.6 ± 3.9
Female (n, %)	41 (46%)
Zygosity (n, MZ/DZ/not twin)	36/26/27

DZ, dizygotic twin; MZ, monozygotic twin; SD, standard deviation.

### 2.2 MEG data acquisition and pre-processing

For controls, we used the HCP rs-MEG data that were acquired using a MAGNES 3600 (4D Neuroimaging, San Diego, CA) MEG system located at the Saint Louis University medical campus. The HCP rs-MEG dataset consisted of three consecutive six-minute runs for each subject. We used the pre-processed rs-MEG data from the HCP S900 data release. Details of pre-processing of the HCP data are described elsewhere (Larson-Prior et al., 2013). Briefly, artifact-contaminated time segments of recordings (which may correspond to head or eye movement) were removed. Noisy MEG channels exhibiting high variance ratio or poor correlation to neighboring channels were identified and removed. The number of channels which were removed due to excessive noise across the controls was 4.0 (avg.), S.D. 2.4. The physiological artifacts (e.g. eye-blinks, muscle artifacts, or cardiac interference) and noise with clear temporal and spectral signatures were identified using independent components analysis (ICA) and then regressed out from the MEG data (Larson-Prior et al., 2013). The sampling rate of the HCP pre-processed MEG data was 508.6 Hz.

The rs-MEG recordings from patients were conducted at the Le Bonheur Children's Hospital using a whole-head MAGNES 3600 MEG system (the same model of MEG system used by HCP) with a sampling rate of 508.6 Hz. Three consecutive 5-minute sessions of resting-state MEG for each patient were used in this study. We used the HCP procedures, mentioned above, for pre-processing of the rs-MEG data of patients by adapting (with slight modification) the pipelines of megconnectome version 3.0 (https://www.humanconnectome.org/software/hcp-meg-pipelines). In pre-processing of the rs-MEG data of patients, in addition to removing artifact-contaminated time segments of recordings (which may correspond to head or eye movement), we visually identified the inter-ictal epileptiform discharges, and then the time segments corresponding to these discharges were removed. We found an average of 13 and S.D. of 3.3 noisy MEG channels across the patients tested. It is noteworthy that the continuous head position monitoring is not possible in the 4D-Neuroimaging MEG system, and thus we did not perform any compensation for head movement.

### 2.3 Functional connectivity and graph measures

The pre-processed rs-MEG data were utilized to calculate the phase-locking value (PLV) as a phase synchronization measure of the brain functional connectivity. The PLV is defined as the absolute value of the mean phase difference between two signals *s*_1_(*t*) and *s*_2_(*t*) (Lachaux et al., 1999):

(1) $$\mathrm{PLV}\left(t;f\right)=\left|E\left[e^{j\left(\varphi_{1}\left(t\right)-\varphi_{2}\left(t\right)\right)}\right]\right|$$

where *φ*_1_(*t*) and *φ*_2_(*t*) are the instantaneous phases of *s*_1_(*t*) and *s*_2_(*t*) in frequency *f*, *E*[.] denotes the expected value, and |.| represents the absolute value of a complex number. The PLV is typically estimated by averaging Eq. (1) over time. The utilization of PLV as a measure of functional connectivity in this study is motivated by these considerations: 1) PLV is able to detect a weak synchronization regime between two areas where the phases of the oscillatory signals in the areas are coupled but the amplitudes of signals may not be (Hramov et al., 2005); 2) our aim was to identify biomarkers to predict VNS seizure outcome in patients with epilepsy, and epilepsy has been historically associated with excessive synchronization of large neuronal populations that can be represented by the PLV (Soriano et al., 2017); and 3) we and other investigators have demonstrated that the values of PLV are an efficient and reliable measure for the functional connectivity in epilepsy and other diseases (Dimitriadis et al., 2015; Elahian et al., 2017).

We calculated the PLV between all pairs of the MEG channels for each run separately using the Fieldtrip toolbox (Oostenveld et al., 2011). We segmented the rs-MEG data of each run into 3-seconds epochs and calculated an average PLV across the epochs in 25 frequency bins from 4 Hz to 30 Hz in steps of 1 Hz. Then we computed an average PLV in theta (4–8 Hz), alpha (8–12 Hz), and beta (12–30 Hz) frequency bands for each run.

The connectivity matrices, corresponding to the PLV between all pairs of MEG channels in three frequency bands, were utilized to compute the graph measures for each run separately. We calculated three global graph measures – modularity, transitivity, and characteristic path length (CPL) – which detect different aspects of functional integration and segregation of the brain network. These graph measures have been widely used to characterize the functional brain network of healthy subjects and patients with neurological or psychiatric diseases (Khazaee et al., 2015; Wu et al., 2017). Modularity is an important graph measure that reflects the neural segregation within a network and represents robustness of the network (Newman, 2006). A network with modular configuration has segregated and non-overlapping communities. Transitivity and the clustering coefficient are measures of segregation of a network that represent strength of connectivity of network nodes to their neighbors (Paldino et al., 2017). The clustering coefficient of a node is defined as the fraction of the node's neighbors that are also neighbors of each other (Watts and Strogatz, 1998). The mean clustering coefficient for a network is defined as the average of the clustering coefficients across all nodes. Transitivity is a normalized variant of the clustering coefficient and does not suffer from the problem that the mean clustering coefficient for a network may be disproportionately influenced by nodes with small neighbors (Rubinov and Sporns, 2010). The CPL of a network is the most commonly used measure of functional integration and is defined as the average shortest path length between all pairs of nodes in the network (Watts and Strogatz, 1998).

**Fig. 1:**
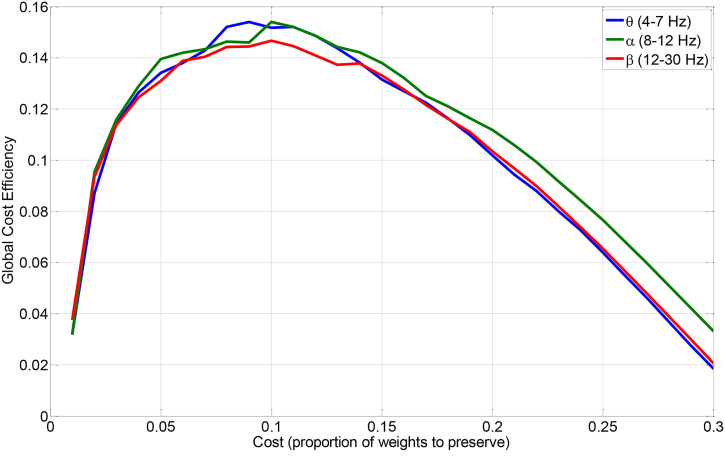
The global cost efficiency (GCE) versus the ratio of the retained strong connections to the total number of connections, defined as cost, in a representative patient. Note that a cost of approximately 10%, i.e. preserving only 10% of the strongest connections, maximizes the GCE.

Similar to our previous studies (Khazaee et al., 2015; Khazaee et al., 2017; Khazaee et al., 2016), we calculated the graph measures after applying an optimal threshold on the connectivity matrixes. By changing the ratio of the retained strong connections to the total number of connections (defined as cost) from 0 to 1, we investigated finding an optimal cost that maximizes the global cost efficiency (GCE) (Bassett et al., 2009). The GCE is defined as the global efficiency minus cost.

(2) $$\mathrm{GCE}=E_{C}-C,\;E_{C}=\frac{1}{n}\sum_{i\in N}E_{i}=\frac{1}{n}\sum_{i\in N}\frac{\sum_{j\in N,j\neq i}d_{\mathrm{ij}}^{-1}}{n-1}$$

where *E*_*C*_ is the global efficiency at cost *C*, *E*_*i*_ is the efficiency of node *i, N* is the set of all nodes in the network, *n* is the number of nodes, *d*_*ij*_ is the shortest path length (distance) between nodes *i* and *j.* It has been shown that the GCE of an economical small-world network has a positive maximum value at an optimal cost. As shown in Fig. 1, we found that a cost of approximately 10%, i.e. preserving only 10% of the strongest connections, maximizes the GCE in our data, and thus we applied this threshold on connectivity matrices before computing the graph measures. We used the average values of the graph measures across three runs in all analyses except in the test-retest reliability analysis, in which we utilized three values of these measures corresponding to three runs.

### 2.4 Statistical analysis for variation of graph measures in three groups

The values of three graph measures (CPL, transitivity, and modularity) in three frequency bands were statistically compared in three groups (non-responders, responders, and controls). First, we compared values of the graph measures in two groups of patients, responders and non-responders. Since approximately one-half of responders and non-responders performed MEG sessions under sedation, we considered sedation status as a binary variable, and a possible confound, in the statistical analysis for patients. For each of nine features (three graph measures in three frequency bands), a two-way analysis of variance (ANOVA) was performed by considering two factors: responding to VNS and sedation status. We utilized the Kolmogorov-Smirnov (K–S) test and found that the ANOVA errors for all graph measures had a normal distribution (the alternative hypothesis of non-Gaussian distributions of the errors was rejected with P > 0.05). However, the results of Levene's test for investigating the equal variances of the graph measures across two groups (responders and non-responders) revealed inequality of the variances in some measures. Since the assumption for equality of variance in ANOVA was not met for some graph measures, we decided to use a non-parametric ANOVA that does not require this assumption. To this end, we used a non-parametric ANOVA based on the bootstrap resampling with replacement (n = 10,000) approach. Since we tested the significance difference of multiple features (three graph measures in three frequency bands), we corrected for multiple comparison by using the false discovery rate (FDR) approach (Benjamini and Hochberg, 1995). The critical value for the FDR was set at the 0.05 level and an FDR-adjusted *P*-value <0.05 was considered statistically significant. Then we investigated a significant difference of the graph measures in controls compared to responders or non-responders by using the Wilcoxon signed-rank test (WSRT). We corrected the WSRT for multiple comparison by again using the FDR approach. As before with the first correction for multiple comparison, the critical value for the FDR was set at the 0.05 level and an FDR-adjusted *P*-value <0.05 was considered statistically significant.

### 2.5 Heritability analysis of rs-MEG graph measures

We used a similar approach detailed in our previous study for heritability analysis of the network topology, derived from the rs-MEG data for controls (Babajani-Feremi, 2017). The calculated global graph measures, as described in Section 2.3, based on the PLV connectivity matrices for 89 control subjects in three frequency bands (theta, alpha, and beta), were used as traits in the heritability analysis. The control subjects had three resting state runs, and an average of the graph measures across these runs was used in the heritability analysis. We used the variance components method implemented in the Sequential Oligogenic Linkage Analysis Routines (SOLAR)-Eclipse software package (http://www.nitrc.org/projects/se_linux) for heritability estimation in the current study. In the variance components method, the total phenotypic variance *σ*_*p*_^2^ in a trait is given by:

(3) $$\sigma_{p}^{2}=\sigma_{g}^{2}+\sigma_{e}^{2}$$

where *σ*_*g*_^2^ and *σ*_*e*_^2^ are the genetic variance due to the additive genetic factors and the variance due to individual-specific environmental effects, respectively.

The rs-MEG global graph measures are traits in this study. Since a complex trait, such as rs-MEG global graph measure, is associated with a large number of environmental factors and genes with small additive effects, a normal distribution with variance of *σ*_*p*_^2^ can model the value of the trait. Considering an assumption that all environmental effects are uncorrelated among family members, the covariance matrix Ω for a pedigree of individuals is defined as:

(4) $$\Omega =2\Phi .\sigma_{g}^{2}+I.\sigma_{e}^{2}$$

where *Φ* is the kinship matrix representing the pairwise kinship coefficients among all individuals and *I* is an identity matrix. The MZ twins share almost 100% of their genes and the differences between them are due to the individual-specific environmental effects. The DZ twins and other siblings share nearly 50% of their genes. The elements of 2*Φ* kinship matrix are equal to 1, ½, and ½ corresponding to the MZ twin pair, the DZ twin pair, and siblings, respectively. The proportion of the phenotypic variance *σ*_*p*_^2^ in a trait that is explained by the additive effects of genes is given by:

(5) $$h^{2}=\sigma_{g}^{2}/\sigma_{p}^{2}$$

The variances *σ*_*g*_^2^ and *σ*_*e*_^2^ were estimated by comparing the covariance matrix predicted by kinship with the observed phenotypic covariance matrix. The significance of heritability of the trait was determined by testing the null hypothesis in which *σ*_*g*_^2^ was constrained to zero. We adjusted the phenotype values for five covariates: sex, age, age^2^, age × sex, and age^2^ × sex. Furthermore, we applied an inverse Gaussian transformation to assure normality of the measurements. The heritability value (*h*^2^) and the significance values (*P*-value) for *h*^2^ and all covariates were reported. The significant *P*-value for *h*^2^ was set to 0.01 (uncorrected).

### 2.6 Reliability of graph measures

Reliability and consistency across all of the rs-MEG-based graph measures is crucial to establish these measures as potential clinical biomarkers. Knowledge of the run-to-run variability of the rs-MEG-based graph measures is an important step toward utilizing these measures in the classification of different categories of patients and healthy controls. In this study, we investigated the test-retest reliability of the rs-MEG-based graph measures by exploring their reproducibility across three resting-state scan sessions, quantified with the intraclass correlation coefficient (ICC) (Shrout and Fleiss, 1979). For calculation of the ICC, we applied a two-way ANOVA to each graph measure corresponding to three rs-MEG scan sessions across either patients or controls. The ICC was defined as:

(6) $$\mathrm{ICC}=\frac{{\mathrm{MS}}_{b}-{\mathrm{MS}}_{E}}{{\mathrm{MS}}_{b}+\left(k-1\right){\mathrm{MS}}_{E}}$$

where *k* = 3 is the number of rs-MEG sessions and *MS*_*b*_ and *MS*_*E*_ are the between-subject mean square and mean square error, respectively. The ICC has a value between 0 (no reliability) and 1 (the highest reliability). Reliability of the graph measures was rated based on the values of ICC as poor (ICC < 0.2), fair (0.2 ≤ ICC < 0.4), moderate (0.4 ≤ ICC < 0.6), good (0.6 ≤ ICC < 0.8), and excellent (0.8 ≤ ICC) (Landis and Koch, 1977).

### 2.7 Classification of responders, non-responders, and controls

The calculated rs-MEG graph measures in theta, alpha, and beta frequency bands were used as input features of a machine learning approach to classify non-responders, responders, and controls. As demonstrated in the Results Section, the modularity and transitivity in theta, alpha, and beta bands were significantly different in responders compared to non-responders. However, the CPL was not significantly different in two groups. Therefore, we did not use the CPL as a feature in the classification. We trained and cross-validated four classifiers using the modularity and transitivity as input features (two features) in three frequency bands: three classifiers corresponding to each of three frequency bands and one classifier corresponding to combination of six features in three frequency bands.

We used the naïve Bayes classifier (NBC) in this study since this classifier is popular, very simple, stable, and fast. Furthermore, the NBC converges quicker than discriminative classifiers, such as logistic regression, and it can be trained with a small dataset. Moreover, classification results of the NBC are not significantly changed by noise and irrelevant features. Additionally, we found that the NBC could outperform other classifiers, e.g. support vector machines (SVM), in our previous study for classifying three groups of subjects based on graph measures extracted from rs-fMRI data (Khazaee et al., 2017). We trained and validated the NBC using the *k*-fold cross-validation (KCV; *k* = 10). We used the KCV in this study, which is one of the most widely used resampling techniques (Anguita et al., 2009), and its estimates for the cross-validation errors nearly agree with the true errors (Braga-Neto and Dougherty, 2004). Performance of the NBC was evaluated using accuracy, sensitivity, specificity, positive predictive value (PPV), and area under receiver operating curve (AUC) measures as criteria.

The number of controls (n = 89) in this study is larger than the number of VNS responders (n = 14) or non-responders (n = 9). Since NBC with unequal group size may lead to biased accuracy of classification, we used a similar approach detailed in our previous paper (Hojjati et al., 2017) and randomly selected 14 out of 89 controls and then evaluated performance of the NBC using those 14 selected controls, 14 VNS responders, and 9 VNS non-responders. The 10-fold cross-validation was repeated 1000 times by randomly selecting 14 out of 89 controls and the average accuracy, sensitivity, specificity, PPV, and AUC were calculated.

## 3 Results

Of the 23 patients with epilepsy included in this study, seizures were reduced by 50% or more at the last follow-up after VNS implantation in 14 (61%) of patients (VNS responders). The last follow-up visits of patients were within 12 to 74 months (30 ± 19 (mean ± SD) months) after VNS implantation. The seizure reduction from pre- to post-VNS treatment was significantly larger in responders than in non-responders (P < 0.0001). In fact, averages of the seizure reduction were 80% and 10% across responders and non-responders, respectively. However, there were no significant differences in gender, age at seizure onset, age at VNS implantation, presence of an MRI lesion, epilepsy type, duration of epilepsy before receiving VNS, follow-up, and pre-VNS seizure frequency in the two groups (P > 0.16) (Table 1). The MEG data were collected in approximately 50% of patients in each group while they were sedated, and there was no significant difference between percentages of sedated patients in the two groups (P > 0.32).

### 3.1 Graph measures in three groups

**Fig. 2:**
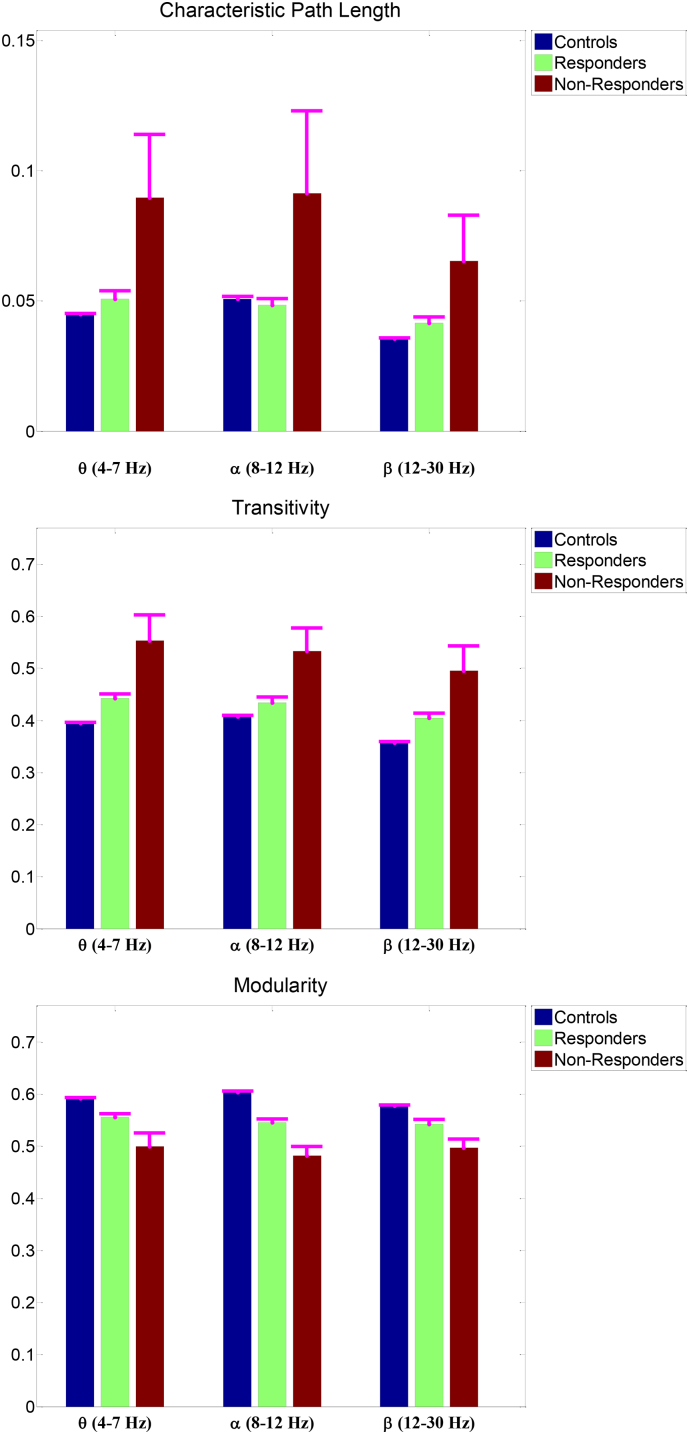
Comparison of the values of the characteristic path length, transitivity, and modularity in three frequency bands in non-responders, responders, and controls.

The CPL, transitivity, and modularity in the three groups (non-responders, responders, and controls) were compared in Fig. 2 and Table 3. We conducted ANOVA to examine differences in graph measures and the effect of sedation in two groups of patients (responders and non-responders). Our results revealed that the modularity in theta, alpha, and beta frequency bands were significantly larger in responders than that in non-responders (P < 0.05, FDR-adjusted). In addition, transitivity values in the three frequency bands were significantly smaller in responders than that in non-responders (P < 0.05, FDR-adjusted). Furthermore, there was no significant difference in CPL in any of three frequency bands in responders vs. non-responders (P > 0.11, FDR-adjusted). Moreover, the effect of sedation was not significant in any of the graph measures in the three frequency bands (P > 0.05).

**Table 3:** The values of three graph measures - characteristic path length, transitivity, and modularity- in non-responders, responders, and controls.

		Values of graph measures in three frequency bands			ANOVA in responders and non-responders			
					Effect of responding to VNS			Effect of sedation
		Controls	Responders	Non-responders	(i/m) Q	P-value	FDR-adjusted P-value	P-value
Characteristic path length	Theta	0.04 ± 0.00	0.05 ± 0.01	0.09 ± 0.07	0.039	0.089	0.115	0.249
	Alpha	0.05 ± 0.01	0.05 ± 0.01	0.09 ± 0.09	0.044	0.142	0.150	0.234
	Beta	0.04 ± 0.00	0.04 ± 0.01	0.07 ± 0.05	0.050	0.150	0.150	0.595
Transitivity	Theta	0.39 ± 0.01	0.44 ± 0.03	0.55 ± 0.14	0.017	0.023	**0.044**	0.212
	Alpha	0.41 ± 0.03	0.43 ± 0.04	0.53 ± 0.13	0.011	0.022	**0.044**	0.051
	Beta	0.36 ± 0.02	0.40 ± 0.03	0.50 ± 0.13	0.033	0.030	**0.044**	0.110
Modularity	Theta	0.59 ± 0.01	0.56 ± 0.02	0.50 ± 0.07	0.022	0.029	**0.044**	0.083
	Alpha	0.60 ± 0.02	0.55 ± 0.03	0.48 ± 0.05	0.006	0.002	**0.014**	0.054
	Beta	0.58 ± 0.02	0.54 ± 0.03	0.50 ± 0.05	0.028	0.029	**0.044**	0.509

These graph measures were calculated based on the PLV, as a measure of rs-MEG functional connectivity, in theta (4–8 Hz), alpha (8–12 Hz), and beta (12–30 Hz) frequency bands. The *P*-values correspond to comparisons of values of the graph measures in two groups of patients (i.e. responders and non-responders). A two-way non-parametric ANOVA based on the bootstrap resampling with replacement (n = 10,000) approach was conducted by considering two factors: responding to VNS and sedation status. Correction for multiple comparison was performed using the FDR approach. An FDR-adjusted *P*-value <0.05 was considered statistically significant (**bold** values).

We investigated significant differences in the graph measures in controls compared to responders or non-responders. Results of WSRT for comparison of the graph measures in controls vs. responders and controls vs. non-responders revealed that the modularity and transitivity in all three frequency bands were significantly different (P < 0.006, FDR-adjusted). In addition, the CPL in theta and beta bands were significantly smaller in controls than responders or non-responders (P < 0.05, FDR-adjusted). However, there were no significant differences between the CPL in alpha in controls vs. responders and controls vs. non-responders (P > 0.09, FDR-adjusted).

### 3.2 Heritability and reliability of graph measures

The heritability estimates for the graph measures in controls are presented in Table 4. The transitivity in three frequency bands were significantly heritable (*h*^2^ > 0.57, P < 0.006). The modularity in alpha and beta bands and CPL in theta and alpha bands were also significantly heritable (*h*^2^ > 0.51, P < 0.005). The covariates sex and age and their interactions (sex, age, age × sex, age^2^, and age^2^ × sex) explained <9% of the phenotypic variance in the graph measures (Table 4). The highest proportion of variance explained by covariates was observed in CPL in the beta band at 8.8% where sex was the most contributed covariate in this feature, though its contribution was not significant (*P* > .03). In fact, all covariates were non-significant (P > 0.01) in explaining the phenotypic variance in the graph measures. Additive genetic factors explained >29% of the residual phenotypic variance in graph measures (*h*^2^ > 0.29). The largest *h*^2^ was observed in the transitivity in the alpha band (*h*^2^ = 0.81, P < 10^−6^) followed by transitivity in the beta band (*h*^2^ = 0.77, P < 1.3 × 10^−5^). The smallest *h*^2^ was observed in modularity in the beta band (*h*^2^ = 0.51, P < 0.005) among all significantly heritable measures.

**Table 4:** The heritability estimates for the graph measures in controls. The graph measures in **bold** font were significantly heritable (P < 0.01).

		*h*^2^	*h*^2^ (*p*-value)	Covariates					
				*p*-value					Variance explained (%)
				Age	Age^2^	Sex	Age×Sex	Age^2^ × Sex	
Characteristic path length	theta	**0.54**	**0.004**	0.45	0.42	0.72	0.16	0.71	0.0
	alpha	**0.54**	**0.002**	0.50	0.86	0.15	0.02	0.07	4.2
	beta	0.36	0.045	0.80	0.15	0.03	0.07	0.09	8.8
Transitivity	theta	**0.57**	**0.006**	0.39	0.89	0.48	0.60	0.24	0.0
	alpha	**0.81**	**7.0** **×** **10**^**−7**^	0.92	0.70	0.22	0.19	0.27	0.0
	beta	**0.77**	**1.2** **×** **10**^**−5**^	0.11	0.79	0.29	0.74	0.21	2.6
Modularity	theta	0.29	0.116	0.04	0.66	0.59	0.12	0.29	0.6
	alpha	**0.54**	**0.002**	0.74	0.47	0.46	0.03	0.84	6.4
	beta	**0.51**	**0.005**	0.32	0.01	0.35	0.22	0.24	6.8

The column “Variance explained (%)” represents the explained percentage of the phenotypic variance by the covariates (Age, Age^2^, Sex, Age × Sex, and Age^2^ × Sex). Additive genetic factors explained the *h*^2^ portion of the phenotypic residual variance. A *P*-value (*h*^*2*^) < 0.01 was considered statistically significant (**bold** values).

The results of the reliability analysis for graph measures are listed in Table 5. The average and median of the ICCs over nine graph features (three graph measures in three frequency bands) and two groups of subjects (controls and patients) were 0.75 and 0.85, respectively. These observations indicate an overall good or excellent test-retest reliability of the rs-MEG graph measures. In fact, 78% of the graph features in patients had an ICC larger than 0.8, which showed an excellent reliability of these measures. In addition, >78% of the graph features in controls had an ICC larger than 0.6, indicating good or excellent reliability of these measures in controls. We observed that the modularity in alpha and beta bands were fairly reliable in patients, and the modularity in these bands had a good reliability in controls. The clinical relevance of fair reliability of the modularity in alpha and beta bands in patients may be utilization of the modularity in theta band, which has good reliability, instead of using alpha and beta bands.

**Table 5:** Test-retest reliability analysis of the graph measures using the ICC approach.

	Characteristic path length			Transitivity			Modularity		
	Theta	Alpha	Beta	Theta	Alpha	Beta	Theta	Alpha	Beta
Controls	0.64	0.85	0.59	0.64	0.85	0.87	0.53	0.75	0.79
Patients	0.95	0.91	0.90	0.95	0.91	0.87	0.85	0.28	0.31

### 3.3 Classification results

We used the graph measures as input features of the NBCs to classify three groups of subjects (i.e., non-responders, responders, and controls). Since the values of CPL in all frequency bands were not significantly different in VNS responders and non-responders, we did not use CPL as an input feature of the NBCs. We trained and cross-validated three classifiers using the modularity and transitivity in theta, alpha, or beta bands. To investigate any improvement in performance of the classifier by integrating features of three frequency bands, the fourth classifier was trained and tested using all six features (two graph measures in three frequency bands).

Performances of four NBCs are compared in Table 6. Accuracies of the classifiers ranged from 71% to 95% which indicates a good performance for all classifiers (accuracy by chance is 33% for a random three group classifier). The average accuracies of three classifiers across three groups in theta, alpha, and beta bands were 85%, 80%, and 82%, respectively; thus, the NBC in theta band outperforms the NBCs in alpha or beta bands. Integration of all features from two graph measures in three frequency bands slightly improved accuracy of the fourth classifier to 87%. In fact, although accuracy of the fourth classifier was larger than that of NBC in theta band, the AUC of the NBC in theta was larger than that of the fourth classifier. It is noteworthy that the fourth classifier consisting of six features provided maximum accuracy, though this classifier has a larger complexity (with six input features) and less generalization compared to the NBC in the theta band (with only two features). Increasing the number of features may result in increasing the accuracy; however, it increases complexity and decreases generality of the model.

**Table 6:** Comparison of performances of four NBCs in classifying non-responders, responders, and controls.

			Confusion matrix									
	Input features		Actual class		Predicted class			Sensitivity	Specificity	PPV	Accuracy	AUC
	*n*	type			NR	R	C					
Classifier 1	2	Transitivity and modularity in theta	Non-responder (NR)	9	5	4	0	0.56	0.94	0.75	0.85	0.86
			Responder (R)	14	2	11	1	0.79	0.78	0.68	0.78	0.85
			Control (C)	14	0	1	13	0.91	0.94	0.90	0.93	0.97
			Weighted average					0.78	0.88	0.78	0.85	0.90
Classifier 2	2	Transitivity and modularity in alpha	Non-responder (NR)	9	5	4	0	0.56	0.89	0.63	0.81	0.83
			Responder (R)	14	3	8	3	0.60	0.78	0.62	0.71	0.83
			Control (C)	14	0	1	13	0.91	0.88	0.83	0.89	0.96
			Weighted average					0.71	0.84	0.70	0.80	0.88
Classifier 3	2	Transitivity and modularity in beta	Non-responder (NR)	9	1	11	2	0.44	0.97	0.87	0.85	0.82
			Responder (R)	14	0	2	12	0.80	0.70	0.62	0.74	0.73
			Control (C)	14	5	4	0	0.86	0.90	0.84	0.88	0.90
			Weighted average					0.73	0.84	0.76	0.82	0.82
Classifier 4	6	Transitivity and modularity in theta, alpha, and beta	Non-responder (NR)	9	5	4	0	0.57	0.94	0.76	0.85	0.83
			Responder (R)	14	2	11	1	0.81	0.80	0.70	0.80	0.84
			Control (C)	14	0	1	13	0.94	0.96	0.93	0.95	0.98
			Weighted average					0.80	0.89	0.80	0.87	0.89

PPV, positive predictive value; AUC, area under receiver operating curve.

Note that the number of controls (n = 89) was larger than the number of responders (n = 14) or non-responders (n = 9). To prevent any bias of NBCs with unequal group size, 14 out of 89 controls were randomly selected, then performance of the NBCs were evaluated using those 14 selected controls, 14 responders, and 9 non-responders. The cross-validation was repeated 1000 times by randomly selecting 14 out of 89 controls and the average number of predicted class, accuracy, sensitivity, specificity, PPV, and AUC across these repetitions are listed in the table.

By looking at the performance of the NBC in theta band in Table 6 it can be observed that this classifier provided a good specificity (≥78%) in three groups and sensitivity (≥79%) in VNS responders and controls. However, this classifier had a moderate sensitivity of 56% in classification of VNS non-responders in that 4 out of 9 non-responders were identified as VNS responders. It is interesting to note that all misclassified non-responders and controls were identified as responders by this classifier. This observation may indicate that VNS responders have an intermediate brain network between controls and non-responders. This is also in concordance with results in Fig. 2, where the graph measures (in all frequency bands) in VNS responders have intermediate values between those in non-responders and controls. These observations may provide some evidence that similarity of the network topology of a patient to that of healthy controls can be an indication for a higher response to VNS treatment.

## 4 Discussion

We investigated whether the rs-MEG network topology before VNS implantation can be used to predict efficacy of VNS treatment in patients with drug resistant epilepsy. We used PLV as a measure of functional connectivity and calculated three graph measures (modularity, transitivity, and CPL) in responders, non-responders, and controls. To investigate whether the rs-MEG graph measures can be considered as potential biomarkers of the network topology, we performed heritability and test-retest analyses on these measures. We found that the graph measures were significantly heritable and had an overall good test-retest reliability, and, thus, they may have a biological basis and can be used as biomarkers. We found that the modularity and transitivity were significantly different in VNS responders and non-responders. We also observed that the modularity and transitivity in controls were significantly different than that in patients. By utilizing the modularity and transitivity as input features of the NBCs, we classified non-responders, responders, and controls with an accuracy of 87%.

Previous electrophysiological studies utilized EEG and investigated reorganization of the brain network after VNS implantation (Fraschini et al., 2014; Fraschini et al., 2013; Bodin et al., 2015). Fraschini et al. used the phase lag index (PLI) between EEG channels after implantation of VNS, and reported a reorganization of the brain network toward a more integrated architecture in responders (Fraschini et al., 2014). Another study investigated the impact of VNS on the synchronicity of interictal EEG rhythms using PLI, and found that VNS responders had significantly smaller synchronization than non-responders (Bodin et al., 2015). Results of these studies provide some evidence that there may be a reorganization of the brain network in responders after VNS treatment; however, these studies have not addressed prediction of efficacy of VNS using baseline (i.e. pre-VNS implantation) measurements. To our knowledge, the current study is the first to investigate prediction of VNS seizure outcome using MEG data acquired before implantation of VNS.

Our results revealed that patients with epilepsy (both VNS responders and non-responders) presented higher transitivity and lower modularity compared to healthy control subjects. Furthermore, this lower segregation pattern (higher transitivity and lower modularity) was also observed in VNS non-responders as compared to responders. The lower segregation of the brain network in patients with epilepsy may indicate a less specialized network in these patients, and describe a network with limited capacity for containing functional processes in specific communities. Brain networks in patients with epilepsy are less robust, thus any module in these networks can be exposed to disruptions from other communities in the brain, which increases the risk of spreading perturbations more easily across brain regions. Lower transitivity and higher modularity in patients responding to VNS, less than that of patients not responding to VNS, may be evidence of effectiveness of VNS in patients with a brain network similar to the network of healthy control subjects.

Our findings are consistent with previous studies reporting functional abnormalities in brain networks of patients with epilepsy compared to healthy control subjects. For instance, Garcia-Ramos et al. observed that the functional brain network in patients with mesial temporal lobe (mTLE) epilepsy presented lower segregation than healthy control subjects, which may be an indication of decreasing robustness and increasing disruption in brain networks of patients with epilepsy (Garcia-Ramos et al., 2016). Another study reported a lower modularity and higher global efficiency in TLE patients compared to healthy controls (Doucet et al., 2015). Although several previous studies investigated alteration of the network topology in patients with epilepsy, there is only one study that investigated the effect of VNS using graph measures, and reported shifting back from an abnormal brain network toward more a normal network in patients responding to VNS (Fraschini et al., 2014). Although it is not possible to directly compare their results with ours, since they used post-implantation VNS data and we utilized pre-implantation VNS data, our results are in agreement with their findings in terms of the effectiveness of VNS in patients with a more complex network similar to the network of healthy control subjects.

A recent study utilized pre-VNS implantation rs-fMRI data and a machine learning approach for classifying responders and non-responders to VNS treatment (Ibrahim et al., 2017). This study utilized correlation coefficients between the time series of the left and right thalami to the ACC and left insula as input features of a SVM classifier, and reported an accuracy of 86% in a two group classification (VNS responders and non-responders). The current study utilized a totally different approach, i.e. we used rs-MEG instead of rs-fMRI, and network topology instead of the raw connectivity, and classified three groups of subjects (non-responders, responders, and controls) with an accuracy of 87%. It is noteworthy that the accuracy reported in (Ibrahim et al., 2017) is similar to that of the current study, but we had a more stringent condition of a three-group classification (accuracy by chance of 33%) compared to a two group classification (accuracy by chance of 50%) in (Ibrahim et al., 2017).

Our results showing heritability of the modularity, transitivity, and CPL (*h*^2^ > 0.51) are in agreement with previous studies demonstrating that the network topology is heritable (Sinclair et al., 2015) (*h*^2^ = 0.42–0.60). Heritability of rs-fMRI functional connectivity in default-mode network and other resting-state networks has also been demonstrated (Fu et al., 2015) (*h*^2^ ~ 0.4). Heritability of functional connectivity has been reported in previous EEG (Schutte et al., 2013) and MEG (Demuru et al., 2017; Colclough et al., 2017) studies. Our heritability results are in agreement with the previous MEG studies, although approaches used by the current study and those of previous studies were different in that the previous studies adopted either an averaging across all pair-wise connectivity values, (Colclough et al., 2017) or investigated whether the pattern of functional or effective connectivity of one monozygotic twin could be utilized to identify the co-twin (Demuru et al., 2017).

In summary, results of the current study and previous studies demonstrated heritability of the network topology, and thus the graph measures derived from rs-MEG functional connectivity may have a biological basis as heritable traits. Establishing the heritability of the rs-MEG graph measures provides critical information necessary before these measures can be considered as biomarkers. Furthermore, we demonstrated that rs-MEG-based graph measures are heritable and that values of these biomarkers were significantly different in VNS responders vs. non-responders. These findings motivate future research for identification of specific candidate genes that may affect a patient's response to VNS treatment.

Our results indicating an overall good test-retest reliability (ICC > 0.6) of the MEG graph measures are in agreement with previous MEG studies (Jin et al., 2011; Deuker et al., 2009). Jin et al. investigated the reliability of rs-MEG nodal network metrics using mutual information (MI) as a measure of functional connectivity between MEG sensors in theta, alpha, beta, and gamma frequency bands (Jin et al., 2011). They reported that the test-retest reliabilities of these metrics ranged from a poor to good level depending on the frequency bands and metrics. Another MEG study investigated the test-retest reliability of graph measures derived from MEG data recorded during an *n*-back working memory task (Deuker et al., 2009). This study utilized MI as a measure of functional connectivity between MEG sensors in different frequency bands and reported a good reliability for most graph metrics in the *n*-back task (ICC ~ 0.62). Previous rs-fMRI functional connectivity studies also investigated reliability of graph measures and reported a poor to good test-retest reliability for these measures (Liao et al., 2013). In summary, results of our study and previous studies indicate reliability and consistency of the rs-MEG-based graph measures; thus, these measures may be considered as potential clinical biomarkers.

### 4.1 Limitations

We recognize the limitations of this study, including a relatively small sample size of VNS responders and non-responders. Although the sample size of patients is small, this study drew from a larger sample of patients (n = 121) who underwent VNS implantation, and is the only study to predict efficacy of VNS using baseline (i.e. pre-VNS implantation) rs-MEG. Furthermore, the sample sizes in previous EEG and fMRI studies for investigating the effect of VNS were also small (n = 10–21) (Fraschini et al., 2014; Fraschini et al., 2013; Bodin et al., 2015; Ibrahim et al., 2017). Moreover, we assessed performance of the NBC using the *k*-fold cross-validation strategy, which is one of the most widely used resampling techniques (Anguita et al., 2009), and its estimates for cross-validation errors are in fair agreement with the true errors (Braga-Neto and Dougherty, 2004). However, we acknowledge that performance of the NBC classifier should also be evaluated in the future on new data that is entirely separate from the training dataset. Another limitation of the current study is that some patients underwent sedation. It is expected that sedation affects the functional connectivity (Barttfeld et al., 2015). However, we think that the effect of sedation is minimized in our study since: 1) sedated and non-sedated patients were distributed evenly in VNS responders and non-responders groups; and 2) we conducted ANOVA to examine the effect of sedation in two groups of patients (VNS responders and non-responders) and found that this effect was not significant in any of the graph measures in three frequency bands (P > 0.05). Another limitation of this study is that the controls were older than the patients, and thus our finding for different values of the rs-MEG graph measures in controls and patients may not be accurate. However, difference in age range of controls and patients does not affect the main findings of this study, specifically: 1) MEG graph measures are heritable (in controls) and they have potential to serve as clinical biomarkers; and 2) transitivity and modularity, as rs-MEG graph measures, are different in VNS responders and non-responders, and values of these measures in baseline (i.e. before VNS implantation) can predict VNS outcome.

## 5 Conclusions

VNS is a low-risk surgical option for patients with drug resistant epilepsy. So far, it is impossible to predict which patients will respond when initiating VNS therapy, and to what extent they will respond. There is a persistent need to find reliable biomarkers that can identify patients who are most likely to respond to VNS and could benefit from this intervention. We found MEG-based network topologies were heritable phenotypes and reliable biomarkers. Our results revealed that transitivity and modularity, calculated using the pre-VNS implantation rs-MEG data, were significantly different in VNS responders compared to non-responders. In addition, transitivity and modularity may be used as biomarkers and input features of a machine learning approach to predict which patients are most likely to respond to VNS.

## Study funding

This study was funded by Le Bonheur Children's Hospital, the Children's Foundation Research Institute, the Le Bonheur Associate Board, Memphis, TN, and LivaNova, Inc., Houston, TX. Data were provided by the Human Connectome Project, WU-Minn Consortium (Principal Investigators: David Van Essen and Kamil Ugurbil; 1U54MH091657) funded by the 16 NIH Institutes and Centers that support the NIH Blueprint for Neuroscience Research; and by the McDonnell Center for Systems Neuroscience at Washington University.
